# ALZHEIMER’S DISEASE GENOMIC, BIOLOGIC, AND BEHAVIORAL PATHWAYS AMONG EXCEPTIONAL LONGEVITY FAMILIES

**DOI:** 10.1093/geroni/igae098.0027

**Published:** 2024-12-31

**Authors:** Mary Wojczynski, Stephanie Cosentino, Nalini Raghavachari

**Affiliations:** Chair; Co-Chair; Discussant

## Abstract

Alzheimer’s disease (AD) is the most common cause of dementia, and its prevalence rates increase sharply in older adults aged 65 and above. Examining whether long-lived families are protected against dementia, and the predictors of dementia within these families, can inform genetic, environmental, and behavioral factors associated with dementia-free survival and the delay of pathological aging. The Long Life Family Study (LLFS), funded by the National Institute on Aging, is an international collaborative study of the genetics and familial components of exceptional longevity and healthy aging. We phenotyped 4,953 individuals from 539 two-generational families (1,727 proband; 3,226 offspring) at baseline (2006-2009), with up to two follow-up in-person visits. These longitudinal, comprehensive in-person visits measured domains of healthy aging, including physical performance, cognition, and blood markers. Extensive genetic analyses were performed using the baseline blood draw, including GWAS chip, linkage analyses, WGS, metabolomics, and transcriptomics. Collectively, this symposium will present novel findings that examined genomic, biological, and behavioral pathways to AD. Specifically, Dr. Xicota will discuss results of the impact of sampling on the limitations of population polygenic risk scores for dementia. Then, Dr. Arbeev will share findings on the longitudinal changes of lysophosphatidylcholine and risk of incident AD. Next, Dr. Cheng will explain results of a multi-omic analysis of the effect of lipids on AD. Lastly, Dr. Gu will discuss analyses of sleep duration in relation to cognition in LLFS. As Discussant, Dr. Nalini Raghavachari from the NIA will share insights and propose future directions for LLFS.

